# Duration of initial prednisolone therapy for first episode of childhood nephrotic syndrome based on time to response

**DOI:** 10.3389/fped.2022.1043285

**Published:** 2022-11-02

**Authors:** Xiaoshan Tang, Qian Shen, Jia Rao, Jing Chen, Xiaoyan Fang, Zhiqing Zhang, Manpreet Grewal, Tej Mattoo, Hong Xu

**Affiliations:** ^1^ Department of Nephrology, Shanghai Kidney Development and Pediatric Kidney Disease Research Center, National Children's Medical Center, Children's Hospital of Fudan University, Shanghai, China; ^2^ Division of Pediatric Nephrology, Department of Pediatrics, Wayne State University School of Medicine, Detroit, MI, United States

**Keywords:** childhood, idiopathic nephrotic syndrome, corticosteroid, duration, time to response

## Abstract

**Background:**

The duration of initial corticosteroid therapy in newly diagnosed Idiopathic nephrotic syndrome (INS) is about 3 months. Our study was designed to test the feasibility of a shorter duration of corticosteroid therapy in newly diagnosed INS who show a quicker response.

**Methods:**

Patients who responded within 10 days (Group A) received 8 weeks of corticosteroid therapy as compared to 12–14 weeks of standard therapy in those who responded between >10 days to 28 days (Group B), and follow up for 52 weeks. The primary endpoint is time to first relapse after treatment completion. (NCT03878914, March 18, 2019)

**Results:**

A total of 33 children with INS were enrolled and the follow-up data were analyzed. The clinical and laboratory characteristics of patients in both groups were similar. No significant difference was found in time to first relapse [65(14.5, 159) days for Group A vs. 28(17, 61.5) days for Group B, *P = 0.371*], the incidence of frequently relapsing nephrotic syndrome [6/18 (33.3%) vs. 5/10(50%), *P = 0.644*] or requirement for alternative immunosuppressant [4/18 (22.2%) vs. 1/10 (10%), *P = 0.769*]. Group A received similar corticosteroid dose compare with Group B (3511 ± 2421 mg/m^2^ vs. 4117 ± 2556 mg/m^2^, *P = 0.524*). Frequency and severity of corticosteroid-related complications was similar in both groups.

**Conclusions:**

The time to first relapse and the number of relapses per patient were comparable between the two groups. However, more patients in Group A relapsed and the mean total dose of prednisolone for the study period was very similar between the two groups.

## Introduction

Idiopathic nephrotic syndrome (INS) is the most common glomerular disease in childhood and corticosteroid therapy is its most effective treatment (1). Although 90% of patients respond by going into complete remission, 70%–80% of patients relapse and, of these, 50% have frequent relapses (FR) or become steroid-dependent (SD) (2). Since inception of corticosteroids in the management of INS by the ISKDC (3), numerous studies have been done with the objective of optimizing response and minimizing the side effects of corticosteroid therapy. Recent studies have demonstrated that the optimal duration of initial corticosteroid therapy should be two to three months and extending it >12 weeks does not significantly improve the outcome (4). The current Kidney Disease: Improving Global outcomes (KDIGO) guidelines recommend a total of 8 or 12 weeks of corticosteroids for all children diagnosed with INS (5). All studies in the past were designed on the principle of single treatment regimen for all patients. No study has ever been done to explore the possibility of treatment duration based on any of the patient characteristics, including time to remission. Patients who respond to corticosteroids within 7 to 10 days are known to have significantly fewer recurrence and a better outcome (6–8). The objective of our study was to test the feasibility of a shorter duration of initial corticosteroid therapy in newly diagnosed patients with INS who show a quicker treatment response.

## Methods

### Study design and patients

We conducted a prospective, open-label, observational cohort study of children with steroid sensitive nephrotic syndrome(SSNS). The study was Initially, designed to enroll a sample size of 64 patients from multiple international study sites (ClinicalTrials.gov, NCT03878914). However, due to the unforeseen disruption caused by COVID-19 pandemic, only one site, Children's Hospital of Fudan University, was able to recruit and allocatedstudy patients and complete follow-up as per the study protocol. Children aged 1 to 19 years old with newly diagnosed SSNS were consecutively included in this study. Patients with high serum creatinine at the time of admission, abnormal labs suggestive of secondary NS, or resistance to initial corticosteroid therapy were excluded from the study. RedCap was used for data collection and management. The Institutional Review Board (IRB) of Wayne State University and Children's Hospital of Fudan University approved and monitored this study (020719MP2F, CFHDU-2019-203, NCT03878914).

**Figure 1:**
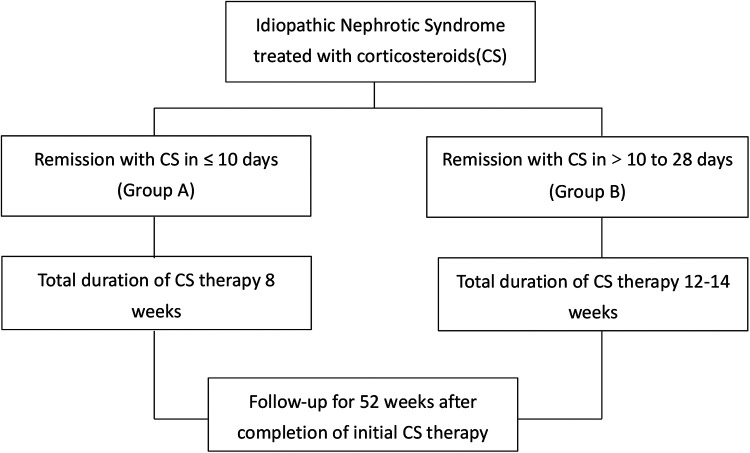
Patient grouping and study design.

As shown in Figure 1, patients were divided into two groups based on time to remission. Patients who achieved remission in ≤10 days (Group A) received prednisolone for a total of 8 weeks where as those who responded between 10 days to 28 days (Group B) received 12–14 weeks of prednisolone. Details on initial steroid therapy and the treatment of relapse(es) for the two groups is shown in Table 1.

**Table 1:** Corticosteroid therapy for initial episode.

Group A (Total duration of therapy 8 weeks)
• 2 mg/kg/day (maximum 60 mg) day for 2 weeks • 1.5 mg (maximum 40 mg) every other day for 2 weeks. • Wean off in 4 weeks
Group B (Total duration of therapy 12–14 weeks)
• 2 mg/kg/day (maximum 60 mg) day for 4 weeks • 1.5 mg (maximum 40 mg) every other day for 4 weeks. • Wean off in 4–6 weeks
**Corticosteroid therapy for a relapse**
• 2 mg/kg/day (maximum 60 mg) until remission • 1.5 mg (maximum 40 mg) every other day for one week followed by continued weaning until discontinued in 6–8 weeks

Definitions used in the study were as follows:

Nephrotic syndrome: Presence of pitting edema with hypoalbuminemia (albumin ≤ 2.5 g/dl or ≤25 g/L) and proteinuria (≥300 mg/dl or 3^+^ on dipstick, or urine protein/creatinine ≥ 2).
Remission: Urine dipstick negative to trace or urine protein/creatinine of <0.2 for 3 days.
Relapse: Protein ≥ 300 mg/dl or 3^+^ on dipstick, or urine protein/creatinine ≥ 0.2 for 3 days.
Frequent relapses: ≥2 relapses within 6 months of initial response or ≥ 4 relapses/12 months.
Steroid dependence: 2 consecutive relapses during CS therapy or within 14 days of discontinuation.
Steroid resistance (Early): No response after 4 weeks of initial CS therapy.
Steroid resistance (Late): No response after 4 weeks of CS therapy for a relapse.

Patients were followed for 52 weeks after completion of initial steroid therapy. Follow-up consisted of outpatient clinic visits and/or video conference at least once every month while on corticosteroids and once every two months when not on corticosteroids. Parents were instructed to examine the first morning urine for protein by dipstick.

The primary study endpoint was time to first relapse after completion of initial steroid therapy. The secondary endpoints included number of relapses during follow-up, number of patients with frequent relapses and or corticosteroid dependence, and the cumulative steroid dose in the two groups.

### Statistical analyses

A two-sample parametric t-test or non-parametric Mann Whitney U test will be employed to examine the mean difference between patients receiving the standard treatment and those receiving prolonged treatment. Categorical data were analyzed with Pearson's chi-square or Fisher's exact test. We used Kaplan-Meier survival curves to visually present the time to first relapse, and compared between groups using a log rank test. Statistical significance will be considered achieved at a *P*-value < 0.05. All statistical procedures will be conducted using SPSS 17.0.

## Results

**Figure 2:**
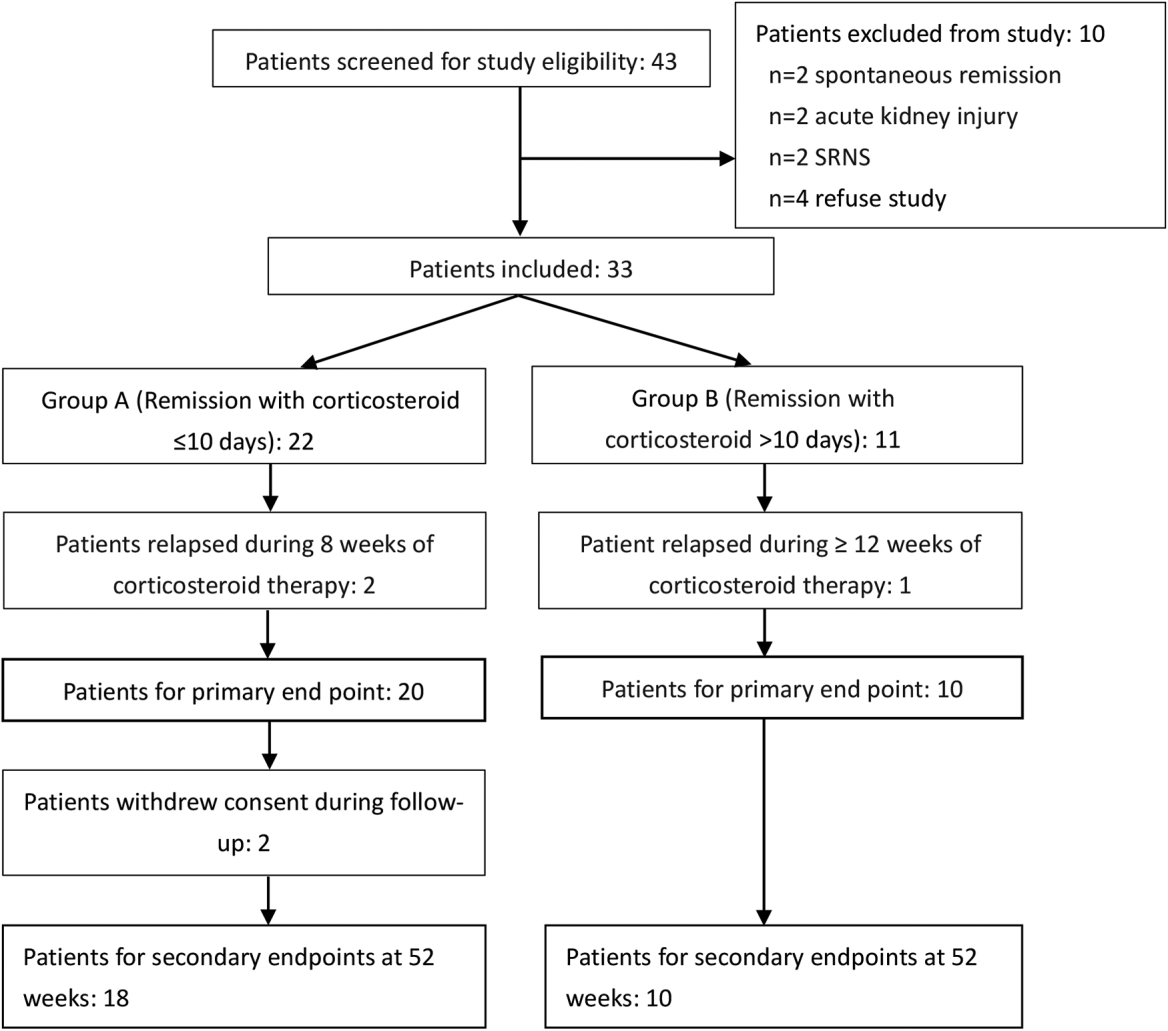
Flow diagram of patients included in the study.

Between August 2019 and July 2020, 43 patients were screened at Fudan University for eligibility and a total of 33 INS children meeting criterion were enrolled in this study. Another 10 patients were excluded due to spontaneous remission (*n* = 2), AKI (*n* = 2), SRNS (*n* = 2), refusing study (*n* = 4). One patient with INS was screened at the Wayne State University but was excluded because of steroid resistance. Three patients (two in Group A and one in Group B) relapsed while on initial corticosteroid treatment. Other two patients in Group A were lost to follow up when they relapsed after completion of initial corticosteroid treatment. Altogether 33 patients were randomized and 28 completed the follow-up (Figure 2).

The median time of response to corticosteroid therapy in 33 patients was 8 (6,11) days; 66.7% (*n* = 22/33) responded within 10 days (Group A) and 33.3% (*n* = 11/33) responded after 10 days (Group B). The clinical and laboratory characteristics of patients in both groups were similar as shown in Table 2. The mean age of disease onset was 3.5 (2.4, 5.4) years; 3.6(2.6, 5.8) years in Group A and 2.8 (1.9, 5.2) years in Group B (*P = 0.911*). Most patients enrolled were boys (*n* = 24,72.7%). The body mass index SDS was similar between two group [0.8(−0.2, 1.4) vs. 1.1(0.0, 1.8), *P = 0.597*] and all patients had normal blood pressure at study enrolment. There was no difference in serum creatinine and albumin between the two groups.

**Table 2:** Baseline characteristics and duration of follow-up.

Characteristics	Group A (*n* = 22)	Group B (*n* = 11)	Total (*n* = 33)
Age at onset in years (Median,IQR)	3.6 (2.6, 5.8)	2.8 (1.9, 5.2)	3.5 (2.4, 5.4)
Boys	16 (72.7%)	8 (72.7%)	24 (72.7%)
**Blood pressure on admission (mm Hg)**			
Systolic (Median, IQR)	91 (90, 100)	91 (85, 100)	91 (90, 100)
Diastolic (Median, IQR)	60 (54, 63)	58 (50, 60)	60 (52, 60)
Body mass index (Median, IQR)	0.8 (−0.2, 1.4)	1.1 (0.0, 1.8)	0.8 (−0.1, 1.5)
**Serum levels on admission (Median, IQR)**			
Albumin (g/L)	15.5 (13.6, 18.1)	17.7 (12.6, 20.9)	16.2 (13.4, 18.7)
Creatinine (umol/L)	28.5 (22.5, 33.0)	23 (20, 29)	25 (22, 32.5)
Days to remission with corticosteroid therapy (Median,IQR)	7 (6,8)	12 (11,15)	8 (6,11)
Duration of follow-up in weeks after completion of 8 (Group A) or 12–14 (Group B) weeks of corticosteroid therapy	52 (*n* = 18) 8,24 (2 withdrew*)	52 (*n* = 10)	

* Urine test, adverse events and growth were collected before withdrew.

**Figure 3:**
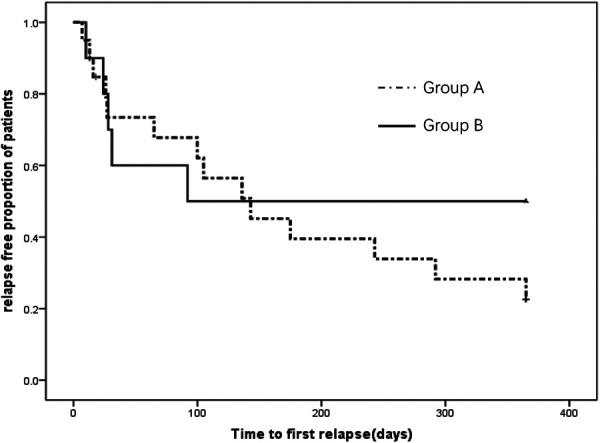
Time to first relapse in participants between groups.

Figure 3 show that the time to first relapse after initial corticosteroid therapy were not significantly different between Group A and Group B patients (Log rank *P = 0.371*). The median time to first relapse was 65 (interquartile range 14.5–159) days for Group A vs. 28 (17–61.5) days for Group B (Table 3). In Group A, 18 of 22 (81.8%) patients had a relapse as compared to 6 of 11 patients (54.5%) in Group B. Included were three patients (2 in Group A, 1 in Group B) who relapsed during initial corticosteroid therapy.

**Table 3:** Primary and secondary outcomes.

Outcome measures	Group A	Group B	*P* Value
**Relapses after completion of corticosteroid therapy**	***N* = 20**	***N* = 10**	* *
• Median time to first relapse (Days)	65 (14.5, 159)	28 (17, 61.5)	
• Total number of relapses	30	13	
• Number of participants who relapsed	16 (80.0%)	5 (50.0%)	*P* = 0.205
• Mean number of relapses/participant	1.5	1.3	*P* = 0.541
**Relapses during initial corticosteroid therapy or during follow-up** *	***N* = 22**	***N* = 11**	
• Total number of relapses	34	16	
• Number of participants who relapsed	18 (81.8%)	6 (54.5%)	*P* = 0.214
• Mean number of relapses/participant	1.54	1.45	*P* = 0.779
**Other clinical outcomes after 52-week follow up**	***N* = 18**	***N* = 10**	
Patients with FRNS/SDNS	6/18 (33.3%)	5/10 (50.0%)	*P* = 0.644
Patients with late steroid resistance	0/18 (0%)	0/10 (0%)	
Patients who received additional immunosuppressant medication	4/18 (22.2%)	1/10 (10%)	*P* = 0.769
Type of alternative immunosuppressant drug:			
Tacrolimus	0/18 (0%)	1/10 (10%)	
Mycophenolate mofetil	2/18 (11.1%)	0/10 (0%)	
Rituximab	2/18 (11.1%)	0/10 (0%)	
**Prednisone dosage (mg/m^2^)**	***N* = 18**	***N* = 10**	
• Mean (SD) total dose of initial prednisolone therapy	1,464 (235.3)	2,143 (812.3)	*P* = 0.0052
• Mean (SD) cumulative prednisolone dose after initial treatment	2,047 (2,416)	1,974 (2,509)	*P* = 0.648
• Mean (SD) total prednisolone dose during the study period	3,511 (2,412)	4,117 (2,556)	*P* = 0.524

FRNS, Frequent relapse nephrotic syndrome; SDNS, Steroid dependence nephrotic syndrome.

* Includes two patients who left the study at 2 and 6 months after initial prednisolone therapy.

Follow-up for 52-weeks was available for 18 and 10 patients in Group A and B, respectively. Two patients from Group A withdrew from the study at 2 and 6 months after completion of initial corticosteroid therapy due to non-adherence to relapse regimen, and they experienced 1 and 3 relapses, respectively, by the time of study withdrawal. A total of 34 and 16 disease recurrences were recorded in Group A and Group B respectively during or after initial corticosteroid therapy. Numbers of relapses per patient were similar (1.54 for Group A vs. 1.45 for Group B, *P* = 0.779), and there was no difference in the proportion of relapsed patients between the tow groups (*P* = 0.214). No significant difference was found in the incidence of FRNS/SDNS [group A 6/18 (33.3%) vs. group B 5/10(50%), *P = 0.644*] or requirement for alternative immunosuppressive treatment [4/18 (22.2%) vs. 1/10 (10%), *P = 0.769*] (Table 3). The delay responders (Group B) developed more SDNS compared with quick responders (Group A), but failed to reach statistical difference (36.3% in Group B vs. 10% in Group A, *P = 0.098*). No patient developed late steroid resistance nephrotic syndrome during follow-up. Overall, the cumulative prednisolone dose per patient in Group A and Group B was similar during the study period (3511 ± 2421 mg/m^2^ for Group A vs. 4117 ± 2556 mg/m^2^ for Group B, *P = 0.524*).

Frequency and severity of corticosteroid-related complications was similar in both groups and it's not obvious overall (Table 4). The most common complication in this study was cushingoid appearance (20% vs. 45.5%, *P = 0.205*), and relieved after corticosteroid reduction. Only one patient with SDNS developed hypertension in Group B. An average of 3 to 4 acute upper respiratory tract infections or other infections occurred per person in both groups. The cohort of this study did not increase the incidence of obesity, but the average BMI *Z*-score of Group A and Group B decreased by 0.98 and 0.53, respectively(*P = 0.106*). In addition, both groups of patients achieved a similar good growth rates (9.0 ± 2.6 vs. 11.1 ± 4.4 cm/year, *P = 0.113*).

**Table 4:** Adverse events and growth in quick and slow responder groups.

	Group A (*n* = 20)	Group B (*n* = 10)	*P* Value
Cushingoid appearance	4 (20%)	5 (45.5%)	*P = 0.205*
Hypertension	0 (0%)	1 (9.1%)	*P = 0.333*
Number of episodes of URI and other infection (mean/pati)	72 (3.3)	38 (3.5)	
Change in BMI *Z*-score	−0.98 ± 1.6	−0.53 ± 1.1	*P = 0.106*
Growth rate (cm/year)	9.0 ± 2.6	11.1 ± 4.4	*P = 0.113*

## Discussion

A growing amount of evidence suggests that the prognosis of children with SSNS is related to their individual characteristics and genetic background. Age onset (7), time to remission (8), immune cell profile (9–11) and genetic background (12, 13) have been implicated to predict the course of disease. Based on these observations, we conducted this prospective cohort study to evaluate the possibility of a shorter course of corticosteroid regimen in patients with a rapid remission after its initiation.

Our study revealed comparable clinical outcomes on primary and secondary clinical endpoints. We found no statistically significant difference between the two treatment groups in the outcome measures: the time to first relapse; number of relapses per patient and number of patients with frequent relapses or corticosteroid dependence during 52 weeks follow up. Patients who received a shorter course (8 weeks) of steroid therapy because of time to remission of ≤10 days had a very similar clinical outcome as compared to those who received a longer duration of steroid therapy (12–14 weeks) because of a time to remission of >10 days. Our results indicate that the time to remission could be one of the factors that might help individualize the duration initial course of corticosteroid therapy in children with SSNS. In subgroup analysis, we found that delayed remission may be a risk factor for SDNS, although it failed to reach statistical difference (*P* = 0.098).

The major concern about steroid therapy in children with INS has been its side effects after prolonged administration. Over the years, the daily dose has remained almost the same but the duration of initial therapy has been researched and debated extensively (14–16). Based on recent data, the Cochrane Review concluded that the optimal duration of initial corticosteroid therapy should be two to three months and extending it >12 weeks does not significantly improve the outcome (4). The most recent Kidney Disease: Improving Global outcomes (KDIGO) guidelines recommend a total of 8 or 12 weeks of prednisone or prednisolone for children diagnosed with INS (5). This one-size-fit-all recommendation for steroid therapy is the result of lack of any studies to define the duration of initial steroid therapy on the basis of individual patient characteristics. Ours is the first study to explore such a possibility by basing the duration of initial steroid therapy on time to response after steroid initiation.

Two patients in Group A and one in Group B relapsed while on initial corticosteroid therapy, which obviously had nothing to do with a shorter course of steroids in the group. After the completion of initial steroid therapy, 82% of patients in Group A had a relapse as compared to 50% in Group B. However, the number of relapses/patient was similar between the two groups. As expected, the mean dose of initial steroid therapy in Group A patients was significantly lower as compared to Group B patients. However, because of more relapses and hence additional steroid therapy, the total dose between the two groups at study conclusion was similar. Furthermore, the proportion of patients who needed additional immunosuppression, other than steroids was more in Group A. These observations undermine our effort to reduce the dose of steroid therapy for initial treatment.

Our study has some limitations; the most important being the number of patients included in the study because of an unforeseen disruption caused by COVID pandemic. Compared with the estimated sample size originally planned, we could randomize only 34 patients, all but one at a single study center in Shanghai, China. The possibility of bias caused by small sample size and uneven patient distribution between the two groups cannot be ruled out. We did not have enough patients for subgroup analysis. Also, one-year follow-up may not have been long enough to evaluate for meaningful outcome differences between the two groups. None the less, ours is the first study to explore the possibility of defining the duration of initial steroid therapy on the basis of patient response to its treatment initiation. We believe that our study will help generate enough interest to explore precision steroid therapy based on patient characteristics rather than currently practice single regimen in all children diagnosed with nephrotic syndrome.

In summary, our study revealed that in patients with rapid remission and a shorter course of steroid therapy, the time to first relapse and the number of relapses per patient were comparable to those with standard treatment. However, more such patients relapsed and the mean total dose of prednisolone for the study period was very similar between the two groups. This is the first study about the possibility of defining initial treatment on individual characteristics and we hope that a larger study with multi-racial population and a longer follow-up will be done in the future to explore individualized treatment in SSNS.

## Data Availability

The raw data supporting the conclusions of this article will be made available by the authors, without undue reservation.
